# Completion lobectomy and central compartment dissection in low-risk patients who had undergone less extensive surgery than hemithyroidectomy

**DOI:** 10.3892/ol.2012.1100

**Published:** 2012-12-28

**Authors:** WEN-BIN YU, YUN-TAO SONG, NAI-SONG ZHANG

**Affiliations:** Department of Head and Neck, Peking University School of Oncology, Beijing Cancer Hospital and Institute, Haidian, Beijing 100142, P.R. China

**Keywords:** papillary thyroid cancer, central compartment, lobectomy

## Abstract

Many low-risk patients with solitary papillary thyroid cancer located in one lobe had undergone surgery that was less extensive than hemithyroidectomy in China. An acceptable completion surgery regimen was suggested for these patients based on our experience. A total of 117 enrolled patients underwent completion surgery. Thirty-two patients had prior tumor resection, 46 patients had prior partial thyroidectomy and 39 patients had prior subtotal thyroidectomy. No neck dissection was performed. Reoperation was scheduled a median of 1.2 months (range, 3 days–6.5 months) after primary surgery for papillary thyroid cancer (PTC). Among the 117 patients, residual tumor was pathologically confirmed in 60 patients, with a residual rate of 51.28%. Among these 60 patients, residual tumor was identified in the thyroid bed alone in 18 patients and in compartment VI alone in 28 patients, while 14 patients exhibited residual tumor in both of these regions. Lymph node metastasis was observed in compartment VI in 42 patients (35.90%), and an average of 6.5 nodes were removed (range, 2–14 nodes for each patient). Additionally, 3.14 positive lymph nodes were removed on average from each of the 42 patients. We conclude that the completion regimen, including the ipsilateral residual lobe, the isthmus and ipsilateral compartment VI (prelaryngeal, pretracheal and paratracheal lymph nodes), is reasonable and acceptable for low-risk patients undergoing surgery that is less extensive than hemithyroidectomy.

## Introduction

Thyroid cancer is one of the 10 most common malignancies in populations worldwide (1,2). Epidemiological data from the USA have demonstrated a 2.4-fold increase in the occurrence of this disease between 1973 and 2002 (3). No global agreement has been reached for thyroid cancer treatment, predominantly due to the fact that it is a long-standing disease with a good prognosis in the majority of patients. A lack of prospective data has meant that practice guidelines are based on large retrospective series and expert opinions. Various authoritative guidelines have been established (4), and these have large differences mainly concerning the extent of thyroidectomy, the extent of lymphadenectomy (central compartment, lateral neck and superior mediastinum) and the post-operative treatment to be performed. The debate regarding the extent of the thyroidectomy to be performed in well-differentiated thyroid cancer focuses on total compared with less than total thyroidectomy, and there continues to be considerable controversy related to unilateral compared with bilateral central compartment dissection (5). The guidelines are dependent on the surgical center, the approach taken by the individual surgeons, the economic situation and the quality of the medical supplies. Recently, the focus of surgical treatment has shifted from increasing the survival rate to decreasing the recurrence rate and improving the quality of life in the treatment of patients with papillary thyroid cancer (PTC) (6) This is due to the excellent prognosis of the disease, which causes operative modalities of thyroid cancer to be more complex.

China has a large area and population size; the number of thyroid cancer patients is extremely high, while the treatment methods vary due to the great disparity in the level of medical care available in different areas, implicating that numerous patients receive an insufficient level of surgery. In China, surgeons managing thyroid cancer include head and neck surgeons, general surgeons and otorhinolaryngologists. Head and neck surgeons in cancer hospitals have received specialized training and are able to perform a standardized thyroidectomy and neck dissection, with the aim of increasing the survival rate, decreasing the recurrence rate and improving the quality of life of thyroid cancer patients. However, the number of thyroid cancer patients treated by head and neck surgeons is limited due to the fact that there are <400 head and neck surgeons in China. Although there are numerous general surgeons in general hospitals in all medical institutions throughout the country, general surgeons are typically not so experienced at managing the recurrent laryngeal nerve, the parathyroid gland and neck dissection, which may lead to tumor tissue being left behind. Addtionally, otorhinolaryngologists have only begun treating thyroid cancer in the past few years; clinical capacity and experience are yet to be accumulated. Radical guidelines in China may lead to further complications and affect the patients’ quality of life. More than 80% of PTC patients belong to the low risk group, accompanied by controversial treatment of thyroidectomy and central compartment dissection. However, there is a great disparity of medical levels in different areas in China. In this study, we represent our characteristical experience of minimally accepted surgical regimen for PTC treatment based on our own experience of salvage surgery on patients with residual tumor and limited surgery efficiency.

## Materials and methods

### Patients and inclusion criteria

Between January 2006 and December 2009, a total of 117 patients diagnosed with papillary thyroid cancer where the tumor was located in one lobe, underwent insufficient primary thyroid surgery that was less extensive than hemithyroidectomy at other institutions. This study was conducted in accordance with the declaration of Helsinki and with approval from the Ethics Committee of Beijing Cancer Hospital and Institute. Written informed consent was obtained from all participants. Patients were suspected to have residual tumor tissue in the ipsilateral thyroid lobe and/or metastasis in the ipsilateral central neck by imaging findings including computed tomography (CT) and high-resolution neck ultrasound examination. The characteristics of patients prior to undergoing completion lobectomy are listed in Table I. The cohort comprised 94 females and 23 males whose median age was 39.3 years and ranged from 16 to 72 years. Thirty-two patients had undergone prior resection of the tumor alone, 46 had undergone prior partial thyroidectomy and 39 had undergone prior subtotal thyroidectomy. No neck dissection was performed. Hoarseness occurred in 17 cases following the initial surgery and vocal cord paralysis was confirmed by fiberoptic laryngoscopy. Reoperations were scheduled a median of 1.2 months (range, 3 days–6.5 months) after primary surgery for PTC. All available clinical, pathological, surgical and diagnostic imaging data were reviewed for accurate staging. Patients undergoing completion operation for PTC were required to meet the following criteria: i) primary tumor size <4 cm; ii) solitary tumor located in one lobe; iii) no palpable or radiological lateral neck lymph node metastases; iv) normal contralateral lobe by ultrasound; v) no distant metastases and vi) no massive extrathyroidal invasion.

### Surgical contents

The extent of surgical resection included the residual affected lobe, the ithmus, the ipsilateral strap muscle adhering to the former surgical area (mainly the sternothyroid muscle and part of the sternohyoid muscle) and ipsilateral compartment VI (the prelaryn geal, pretracheal and paratracheal lymph nodes).

### Surgical approach

**Figure 1:**
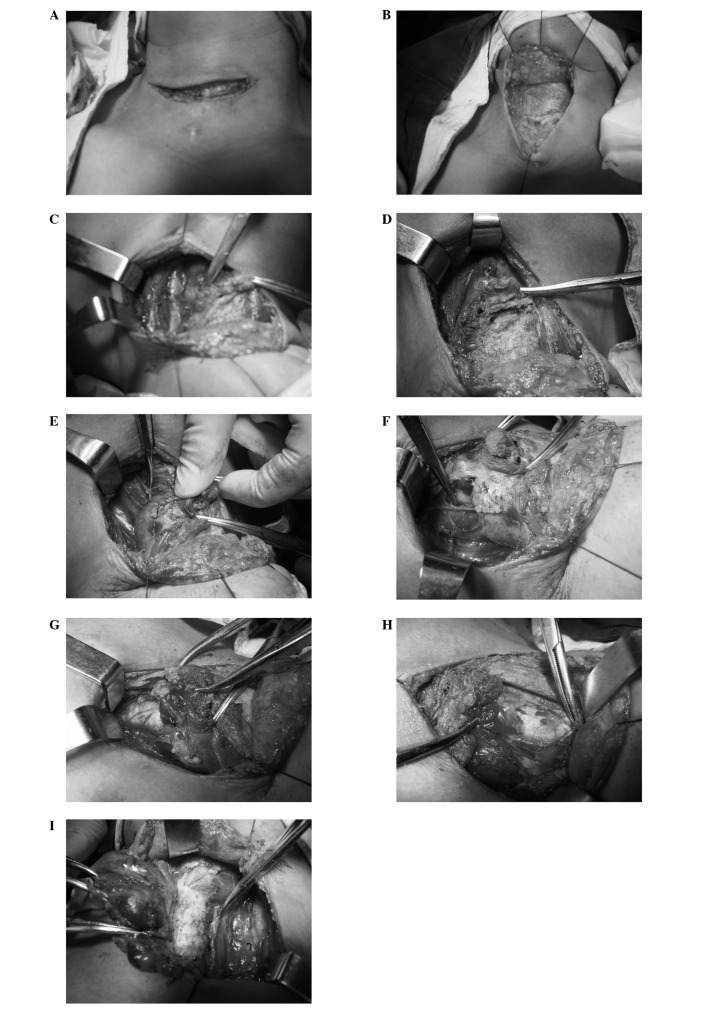
Surgical approach. (A) A low collar incision is conducted on the previous incision, with excision of the previous scar. (B) A subplatysmal dissection is elevated superiorly and inferiorly, elevating the neck flaps. (C) The carotid artery is dissected from the level of the thyroid cartilage down to the clavicle. (D) The infrahyoid strap musculature is inferiorly resected at the level of the sternal notch. (E) The recurrent laryngeal nerves are identified most inferiorly in the tracheoesophageal groove. (F) The nerves are meticulously microdissected from the clavicle up to the scar tissue region. (G) Pretracheal tissues are also dissected to connect with the residual lobe. (H) The infrahyoid strap musculature is resected superiorly at the level of the cricoid cartilage. (I) The lymph-fatty tissues in compartment VI, the ipsila teral residual lobe and the ipsilateral strap muscle are dissected according to the continuous *en bloc* principle.

A low collar incision was made on the previous incision, with excision of the previous scar and extension into the lower neck creases to the anterior margin of the sternocleidomastoid muscle. A subplatysmal dissection was elevated superiorly and inferiorly, elevating the neck flaps. The inferior neck flap was elevated well below the sternal notch. The infrahyoid strap musculature was cut off at the levels of the cricoid cartilage, superiorly and the sternal notch, inferiorly. The carotid artery was dissected from the level of the thyroid cartilage down to the clavicle. The recurrent laryngeal nerves were identified in areas previously lacking or with minimal dissection, thus the laryngeal nerves were identified most inferiorly in the tracheoesophageal groove. The nerves were meticulously microdissected from the clavicle to the scar area. The nerves were pulled outwards, and the lymph nodes and adipose tissue in the tracheoesophageal groove were dissected to connect with the residual lobe. Pretracheal tissues were also dissected to connect with the residual lobe. Finally, the nerves in the scar tissue regions were dissected into their laryngeal inlet, and the frequently arborized laryngeal branches were identified and completely spared. The lymphatic fatty tissues in compartment VI and around the lobe, the ipsilateral residual lobe and the ipsilateral strap muscle were cleared away according to the continuous *en bloc* principle (Fig. 1). When the parathyroid tissue was identified to be distinct from the resected tissue, it was immediately finely minced and then the frozen section was pathologically confirmed. Parathyroid glands confirmed by pathological analysis were considered for autotransplantation into the sternocleidomastoid muscle.

## Results

### Overall tumor residue

Among the 117 patients, pathological analysis confirmed the presence of residual tumor in 60 patients, with a residual rate of 51.28%. Among these 60 patients, 18 had residual tumor in the thyroid bed alone, while 28 had residual tumor in compartment VI alone, and 14 had residual tumor in both the thyroid bed and compartment VI (Table II).

### Lymph node metastasis in compartment VI

All 117 patients underwent compartment VI dissection. Lymph node metastasis was confirmed in compartment VI in 42 patients (35.90%), with an average of 6.5 nodes removed (range, 2–14 nodes for each patient). In addition, an average of 3.14 positive lymph nodes were confirmed in each of these patients.

### Complications

The cervical part of the recurrent laryngeal nerves was fully dissected in all 117 patients. Among the 17 patients with hoarseness following initial surgery in other institutions, 13 patients had undergone subtotal thyroidectomy and 4 patients had undergone partial thyroidectomy. Investigation during reoperation revealed that the recurrent laryngeal nerves had been completely resected in 12 patients. Of these nerves, 8 had been resected near the interaction with the inferior thyroid artery and 4 had been resected within 1 cm of the laryngeal inlet, while 5 had been sutured near the laryngeal inlet.

Among the 100 patients without recurrent laryngeal nerve damage following initial surgery, 6 patients developed hoarseness after reoperation. Other complications included hematoma in two patients, wound infection in one patient and transient hypoparathyroidism in two patients.

### Survival

Patients were followed up for ≥18 months (range, 18–66 months). Seven patients (5.98%) developed recurrence following reoperation. The recurrence was observed in the contralateral thyroid gland in two patients, in the contralateral tracheoesophageal groove in two patients and in the ipsilateral lateral neck in three patients. All patients with recurrence received salvage surgery and no mortality was recorded.

## Discussion

The management of papillary thyroid carcinoma continues to be an area of debate and controversy (6,7), which includes the extent of thyroidectomy, the extent of lymphadenectomy and the type of postoperative treatment to be performed. The debate concerning the extent of thyroidectomy in well-differentiated thyroid cancer focuses on total versus less than total thyroidectomy. As the serum thyroglobulin level may be used as a marker of recurrence, radioactive iodine (RAI) ablation may be performed as an adjuvant therapy and multifocality is often observed (8,9). Western countries usually routinely perform total thyroidectomy followed by RAI ablation and thyroid stimulating hormone (TSH) suppression as the standard treatment (4,10,11), particularly in patients with a tumor size >1 cm. However, total thyroidectomy is not accepted by all thyroid surgeons worldwide, particularly for low-risk patients. In the past decade, the majority of patients with primary tumors <4 cm in size, no lymph node metastases and a normal contralateral lobe determined by ultrasound examination have been treated with thyroid lobectomy alone, at the Memorial Sloan-Kettering Cancer Center (12). This indicates that lobectomy has been accepted by Western surgeons in low-risk patients with PTC. Studies from Japan and China, which are both countries with a large population size, demonstrate that the majority of low-risk patients with papillary thyroid carcinoma become disease-free when they undergo surgery that is less extensive than total thyroidectomy (13). Additionally, recurrent laryngeal nerve paralysis and hypoparathyroidism may be decreased, L-thyroxine may not be administered throughout the patient’s life and the recurrence rate of contralateral thyroid gland of patients who underwent hemithyroidectomy has been demonstrated to be low (only 1% for T1NOMO patients in Japan) (14). Multifocalities have been detected in the range of 15.0–43.8% (8,9,15). However, for patients with a solitary tumor detected by imaging findings (the minimal focus size typically detected is ∼3 mm) and with multifocalities detected by pathological examination, whether it is clinically beneficial to treat these small pathological focuses remains unclear. A study from Japan concerning microcarcinoma helps us to understand multifocalities detected by pathological examination from a different perspective (16). No size changes were observed in 70% of microcarcinomas, while a size enlargement (>3 mm) was identified in only 6.7% of the microcarcinomas and no distant metastasis or death of the thyroid carcinoma were observed after a 5-year follow-up. The authors suggested that observation of PMC is a reasonable strategy for microcarcinoma incidentally detected by imaging studies. Therefore, microcarcinoma detected by pathology is of less clinical significance than that detected by imaging.

The treatment results of total thyroidectomy and hemithyroidectomy remain controversial for low-risk PTC (15,17). A study by Shah *et al* demonstrated that no differences in cause-specific survival, local recurrence, regional recurrence and distant metastasis at 20 years were observed between low-risk patients undergoing lobectomy and total thyroidectomy (18). Moreover, data from China have demonstrated that good survival rates and low morbidities were achieved, and that lobectomy was suggested to Chinese surgeons (19). Medical institutions are widely distributed in China, with a great disparity in the medical levels of different regions. Radical surgery, as with Western countries, may cause further complications and decrease patients’ quality of life. Therefore, according to experiences in China and to other data, for low-risk PTC, lobectomy does not decrease survival and increase recurrence rates, while it decreases complications and is suitable for use in China. Due to the aforementioned reasons, our center considers lobectomy to be the initial treatment for low-risk PTC with a solitary tumor located in one lobe. Additionally, we now perform completion lobectomy in such patients who have undergone surgery that is less extensive than lobectomy, particularly for patients who undergo nodulectomy alone for benign disease. In our study, 32 out of 117 patients who underwent surgery less extensive than lobectomy were observed to have residual tumor in the left ipsilateral lobe, which indicates that it was necessary for those patients to undergo completion surgery.

Compartment VI dissection is another debated issue in PTC treatment. The value of compartment VI dissection is determined by whether it improves survival or decreases recurrence rates; whether it increases the risk of hypoparathyroidism and recurrent laryngeal nerve damage; and whether reoperation in compartment VI increases the risk of hypoparathyroidism and recurrent laryngeal nerve damage (20).

Certain authors argue that compartment VI metastasis has no effect on long-term survival (8,21). Sugitani and Fujimoto reported that patients with compartment VI metastasis have a poorer prognosis than those without metastasis (22. Vini *et al* confirmed that compartment VI metastasis may have prognostic implications, particularly for those patients >45 years of age (12). Current evidence shows a potential decrease in local recurrence and a possible survival benefit are associated with compartment VI dissection (23). Compartment VI dissection may cause a higher rate of permanent hypoparathyroidism and unintentional permanent recurrent laryngeal nerve injury, while experienced thyroid surgeons are able to decrease the rate of complications. It has been demonstrated that a higher rate of hypocalcemia and nerve damage is expected in reoperation in patients with compartment VI recurrence (24). Additionally, the diagnostic accuracy of pre-operative imaging for compartment VI metastasis is very low; ultrasonography is the most important examination and is confirmed to have a high positive predictive value and sensitivity, but a low negative predictive value and specificity (25). For the reasons listed previously, more surgeons are considering compartment VI dissection in the initial treatment for PTC patients (26–29).

There continues to be considerable controversy related to unilateral or bilateral central compartment dissection (5). In our hospital, we take unilateral central compartment dissection (prelaryngeal, pretracheal and paratracheal lymph nodes) to be the standard treatment for patients with PTC located in one lobe. The reasons for this are as follows: i) recurrent laryngeal nerve dissection is the basic step for both lobectomy and unilateral central compartment dissection, and tissues in these two compartments may be resected as a whole body according to continuous *en bloc* and tumor-isolating principles; ii) complications, including hypoparathyroidism and bilateral recurrent laryngeal nerve injury, may be decreased or possibly avoided; iii) positive lymph nodes are mainly distributed in the unilateral central compartment of the tumor, and the risk of reoperation in the contralateral central compartment would be low as no dissection would have occurred in the initial surgical procedure.

In China, treatment of the central compartment is not satisfactory, as numerous surgeons in Chinese community hospitals do not consider metastasis in the central compartment to be an important part of PTC treatment, and positive lymph nodes in the central compartment are typically ignored, which is a potential risk for recurrence. In the present study, none of the 117 patients underwent compartment VI dissection, while 42 patients had confirmed lymph node metastasis, which strongly indicates the need for routine compartment VI dissection.

Due to the evidence discussed previously, in our department, we now perform salvage surgery, which includes the ipsilateral residual lobe, the isthmus and ipsilateral compartment VI (the prelaryngeal, pretracheal and paratracheal lymph nodes), in low-risk patients undergoing surgery that is less extensive than hemithyroidectomy. The key problem is how to perform the surgery according to *en bloc* and tumor-isolating principles following confirmation of the extent of salvage surgery required. The surgical approach that we adopted based on our own experience and understanding, and that has not been demonstrated in other studies, has been introduced in this study (detailed in Surgical approach). This surgical approach achieves *en bloc* resection of the ipsilateral residual lobe, the residual tumor, the isthmus, ipsi-lateral compartment VI and the contaminated strap muscles, according to the tumor-isolating principles that have become our standard approach to salvage surgery.

## Figures and Tables

**Table I:** Characteristics of patients prior to completion surgery.

Characteristics	No. (%)
Gender	
Female	94 (80)
Male	23 (20)
Initial surgery	
Tumor resection	32 (27)
Partial thyroidectomy	46 (39)
Subtotal thyroidectomy	39 (34)
Lymph node dissection	
Compartment VI	0 (0)
Other compartment	0 (0)
Complications	
Recurrent laryngeal nerve damage	17 (15)
Hypoparathyroidism	0 (0)
TNM stage at initial surgery	
T1	43 (37)
T2	74 (63)
N0	0 (0)
N1	0 (0)

**Table II:** Overall tumor residue.

Location	No. (%)
Thyroid bed	18 (30)
Compartment VI region	28 (47)
Thyroid bed and compartment VI region	14 (23)

## References

[1] Cooper DS, Doherty GM, Haugen BR (2006). Management guidelines for patients with thyroid nodules and differentiated thyroid cancer. Thyroid.

[2] Sundram F, Robinson BG, Kung A (2006). Well-differentiated epithelial thyroid cancer management in the Asia Pacific region: a report and clinical practice guideline. Thyroid.

[3] Davies L, Welch HG (2006). Increasing incidence of thyroid cancer in the United States, 1973–2002. JAMA.

[4] Cooper D, Doherty G, Haugen B (2009). Revised American thyroid association management guidelines for patients with thyroid nodules and differentiated thyroid cancer. Thyroid.

[5] Shaha AR (2009). Prophylactic central compartment dissection in thyroid cancer: a new avenue of debate. Surgery.

[6] Grant CS, Stulak JM, Thompson GB, Richards ML, Reading CC, Hay ID (2010). Risks and adequacy of an optimized surgical approach to the primary surgical management of papillary thyroid carcinoma treated during 1999-006. World J Surg.

[7] Gary LC, Shellenberger TD, Ginsberg LE (2009). Approach and safety of comprehensive central compartment dissection in patients with recurrent papillary thyroid carcinoma. Head Neck.

[8] Wada N, Duh QY, Sugino K (2003). Lymph node metastasis from 259 papillary thyroid microcarcinomas. Ann Surg.

[9] Chow SM, Law SC, Chan JK, Au SK, Yau S, Lau WH (2003). Papillary microcarcinoma of the thyroid-prognostic significance of lymph node metastasis and multifocally. Cancer.

[10] Takami H, Ito Y, Okamoto T, Yoshida A (2011). Therapeutic strategy for differentiated thyroid carcinoma in Japan based on a newly established guideline managed by Japanese Society of Thyroid Surgeons and Japanese Association of Endocrine Surgeons. World J Surg.

[11] Sywak M, Pasieka JL, Oglivie T (2004). A review of thyroid cancer with intermediate differentiation. J Surg Oncol.

[12] Vini L, Hyer SL, Marshall J, A’Hern R, Harmer C (2003). Long-term results in elderly patients with differentiated thyroid carcinoma. Cancer.

[13] Ito Y, Miyauchi A (2009). Prognostic factors and therapeutic strategies for differentiated carcinomas of the thyroid. Endocr J.

[14] Ito Y, Masuoka H, Fukushima M (2010). Excellent prognosis of patient with solitary T1N0M0 papillary thyroid carcinoma who underwent thyroidectomy and elective lymph node dissection without radioiodine therapy. World J Surg.

[15] Falvo L, D’Ercole C, Sorrenti S (2002). Papillary microcarcinoma of the thyroid gland: analysis of prognostic factors including histological subtype. Eur J Surg.

[16] Ito Y, Uruno T, Nakano K (2003). An observation trial without surgical treatment in patients with papillary microcarcinoma of the thyroid. Thyroid.

[17] Shaha AR, Shah JP, Loree TR (1997). Low-risk differentiated thyroid cancer: the need for selective treatment. Ann Surg Oncol.

[18] Shah JP, Loree TR, Dharker D, Strong EW (1993). Lobectomy versus total thyroidectomy for differentiated carcinoma of the thyroid: a matched-pair analysis. Am J Surg.

[19] Head and Neck Surgery Group, Editorial Board of Chinese Journal of Otorhinolaryngology Head and Neck Surgery (2011). Discussion on clinical guideline of differentiated thyroid carcinoma. Zhonghua Er Bi Yan Hou Tou Jing Wai Ke Za Zhi.

[20] White ML, Gauger PG, Doherty GM (2007). Central lymph node dissection in differentiated thyroid cancer. World J Surg.

[21] Shaha AR, Tuttle RM, Shah JP (2007). Papillary microcarcinoma of the thyroid. J Surg Oncol.

[22] Sugitani I, Fujimoto Y (1999). Symptomatic versus asymptomatic papillary thyroid microcarcinoma: a retrospective analysis of surgical outcome and prognostic factors. Endocr J.

[23] Gemsenjäger E, Perren A, Seifert B, Schüler G, Schweizer I, Heitz PU (2003). Lymph node surgery in papillary thyroid carcinoma. J Am Coll Surg.

[24] Lim DJ, Baek KH, Lee YS (2007). Clinical, histopathological, and molecular characteristics of papillary thyroid microcarcinoma. Thyroid.

[25] Ito Y, Tomoda C, Uruno T (2005). Ultrasound-detectable and anatomopathologically-detectable node metastasis in the lateral compartment as indicators of worse relapse-free survival in patients with papillary thyroid carcinoma. World J Surg.

[26] Ito Y, Jikuzono T, Higashiyiama T (2006). Clinical significance of lymph node metastasis of thyroid papillary carcinoma located in one lobe. World J Surg.

[27] Shindo M, Wu JC, Park EE, Tanzella F (2006). The importance of central compartment elective lymph node excision in the staging and treatment of papillary thyroid cancer. Arch Otolaryngol Head Neck Surg.

[28] Machens A, Hauptmann S, Dralle H (2009). Lymph node dissection in the lateral neck for completion in central node positive papillary thyroid cancer. Surgery.

[29] Swyak M, Cornford L, Roach P, Stalberg P, Sidhu S, Delbridge L (2006). Routine ipsilateral level VI lymphadenectomy reduces postoperative thyroglobulin levels in papillary thyroid cancer. Surgery.

